# A case of intraplacental gestational choriocarcinoma; characterised by the methylation pattern of the early placenta and an absence of driver mutations

**DOI:** 10.1186/s12885-019-5906-8

**Published:** 2019-07-29

**Authors:** Philip Savage, David Monk, Jose R. Hernandez Mora, Nick van der Westhuizen, Jennifer Rauw, Anna Tinker, Wendy Robinson, Qianqian Song, Michael J. Seckl, Rosemary A. Fisher

**Affiliations:** 1 Trophoblastic Tumour Screening & Treatment Centre, Charing Cross Hospital Campus of Imperial College, London, UK; 2 BCCA, Victoria, BC Canada; 3 Imprinting and Cancer Group, Cancer Epigenetic and Biology Program (PEBC), Bellvitge Biomedical Research Institute (IDIBELL), L’Hospitalet de Llobregat, Barcelona, Spain; 4 Department of Medical Genetics, University of British Columbia, Vancouver, BC Canada; 5 State Key Lab of Molecular Oncology, Laboratory of Cell and Molecular Biology, National Cancer Center, Beijing, China; 6 Department of Surgery and Cancer, Imperial College , London, UK

**Keywords:** Oncogenesis, Trophoblast, Placenta, Choriocarcinoma, Methylation, Epigenetics, Pregnancy, Chemotherapy

## Abstract

**Background:**

Gestational choriocarcinoma is a rare malignancy believed to arise from the trophoblast cells of the placenta. Despite the frequently aggressive clinical nature, choriocarcinoma has been routinely curable with cytotoxic chemotherapy for over 50 years. To date little is known regarding the route to oncogenesis in this malignancy.

**Methods:**

In a case of intraplacental choriocarcinoma, we have performed detailed genetic studies including microsatellite analysis, whole genome sequencing (WGS) and methylation analysis of the tumour and surrounding mature placenta.

**Results:**

The results of the WGS sequencing indicated a very low level of mutation and the absence of any driver mutations or oncogene activity in the tumour. The methylation analysis identified a distinctly different profile in the tumour from that of the mature placenta. Comparison with a panel of reference methylation profiles from different stages of placental development indicated that the tumour segregated with the first trimester samples.

**Conclusions:**

These findings suggest that gestational choriocarcinoma is likely to arise as a result of aberrations of methylation during development, rather than from DNA mutations.

The results support the hypothesis that gestational choriocarcinoma arises from a normally transient early trophoblast cell. At this point in development this cell naturally has a phenotype of rapid division, tissue invasion and sensitivity to DNA damaging chemotherapy that is very similar to that of the mature choriocarcinoma cell.

**Electronic supplementary material:**

The online version of this article (10.1186/s12885-019-5906-8) contains supplementary material, which is available to authorized users.

## Background

Gestational Trophoblastic Neoplasia (GTN) are a group of rare conditions that arise from the cells of conception. The most frequent forms are the pre-malignant genetically abnormal complete and partial hydatidiform moles which arise from an androgenetic or triploid dispermic conceptus respectively. The clinically more complex malignant diagnoses of gestational choriocarcinoma, placental site trophoblastic tumour (PSTT) and epithelioid trophoblastic tumour (ETT), whilst on occasion can arise from a molar pregnancy, more usually each arise from pregnancies that have the normal genetic complement [1].

Clinically gestational choriocarcinoma is frequently characterised as an invasive, fast growing and aggressive cancer. Presentation during the causative pregnancy is rare and most cases are identified some months or years following the causative pregnancy often as a result of symptoms from distant metastatic spread. The overall incidence of gestational choriocarcinoma is estimated at 1 case per 50,000 pregnancies and aside from increasing maternal age does not appear to have any other significant risk factors [2].

Despite this rarity and the rapidity of cell growth, gestational choriocarcinoma has been curable with cytotoxic chemotherapy since the 1950s and in modern series the overall cure rate now approaches 95% [3, 4].

Conventionally malignancies characteristically arise from previously normal cells that develop the malignant phenotype, including uncontrolled growth and invasion, as a result of a number of DNA mutations [5]. The normally transient early trophoblast cells have a phenotype, including rapid growth, local invasion and stimulation of angiogenesis that is similar to that of a malignant cell. As a result, it has been suggested that gestational choriocarcinoma may not necessarily represent a classical mutation based malignant transformation. Instead these tumours could arise due to the persistence of early trophoblast cells, which have failed to either mature or undergo apoptosis [6].

In contrast to most common malignancies, gestational choriocarcinoma is frequently managed based on a clinical diagnosis without a biopsy and therefore tumour samples of sufficient quantity to permit detailed genetic or methylation analysis are exceptionally rare. As a result, no previous whole genome sequencing or detailed methylation studies have been reported for this rare diagnosis.

Gestational choriocarcinoma is thought to originate from the cytotrophoblast and syncytiotrophoblast cells that develop into the placenta. Since most cases are not diagnosed until months or years after the end of the pregnancy, in the large majority of cases of choriocarcinoma the placenta has been discarded before the diagnosis is clinically apparent. However, in very rare cases an intra-placental choriocarcinoma is noted within the placenta at delivery or on pathological examination. This is therefore the earliest clinical time point for diagnosis of a choriocarcinoma and such material provides a unique opportunity to study the genetic or epigenetic changes that could drive the malignant behaviour of this tumour prior to any additional later acquired changes. Interestingly, even in these ‘early’ choriocarcinomas approximately 50% are already associated with metastatic spread. Fortunately, with modern chemotherapy the cure rate for women with these rare intraplacental choriocarcinomas approaches 100% [7].

Here we have investigated the potential contribution of genetic and epigenetic changes to the pathogenesis of this rare malignancy, using whole genome sequencing and methylation analysis from a recent case of intraplacental gestational choriocarcinoma.

### Case presentation

After an uneventful second pregnancy a 41-year-old caucasian woman delivered a 39-week female baby via emergency caesarean section for fetal distress. At delivery the baby was unwell and despite resuscitation, sadly died 19 h post-delivery.

**Fig. 1:**
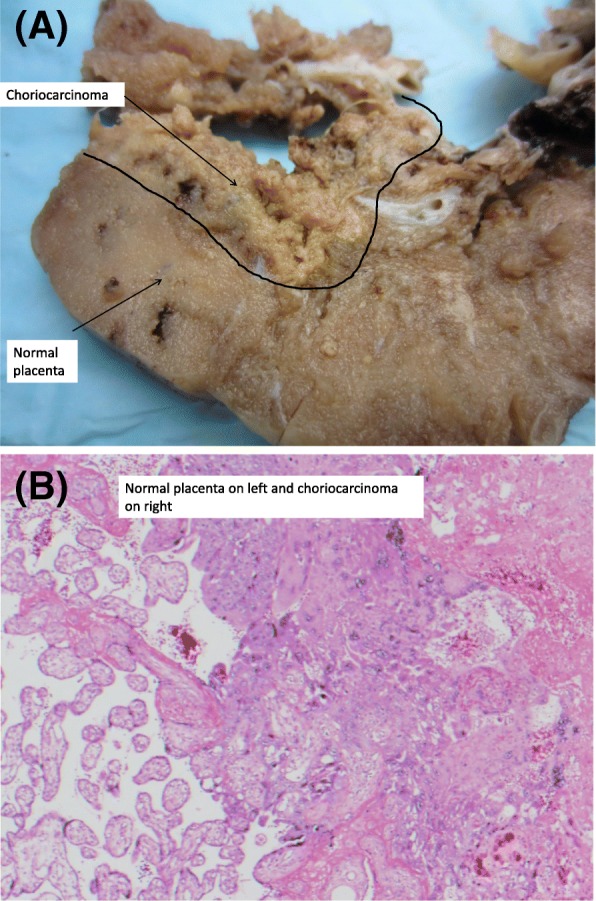
Pathology of intraplacental choriocarcinoma. **a** Gross morphology of choriocarcinoma within the normal placenta. **b** Haematoxylin and eosin stained section of tumour (right) and surrounding normal placenta (left)

Macroscopic examination of the placenta demonstrated a 4 cm abnormality (Fig. 1a). On histopathological review the diagnosis of gestational choriocarcinoma was made based on the morphological appearance of sheets of atypical mononucleated cytotrophoblast admixed with multinucleated syncytiotrophoblast with associated haemorrhage and necrosis (Fig. 1b). A fetal post mortem was not performed.

In response to the diagnosis of choriocarcinoma, the patient was assessed for the presence of metastatic disease with an MRI scan of the head and pelvis and a CT of the chest and abdomen and serial serum human chorionic gonadotrophin (hCG) monitoring. The imaging showed no evidence of distant disease. The hCG level immediately post-partum was 202,499 IU/L and then fell sequentially with the expected 1–2 day half-life for complete removal of tumour reaching the normal range approximately 30 days post-delivery. On follow up the patient has remained well, with a normal serum hCG for more than 3 years and the chance of relapse is now remote.

## Methods

### Genetic investigations

With the patient’s consent and in keeping with the institutional ethics policy further genetic analysis of the placenta and tumour were performed.

### Preparation of genomic DNA

With reference to a consecutive haematoxylin and eosin stained section, tumour tissue and surrounding normal placenta were micro-dissected independently from unstained formalin-fixed paraffin-embedded (FFPE) sections. DNA was prepared from dissected tissue using a QIAmp DNA FFPE Tissue Kit (Qiagen, UK) and the DNA quantified using a Picogreen dsDNA quantitation kit (Life Technologies, UK).

### Fluorescent microsatellite genotyping

To exclude the possibility that the tumour could have been either a metastasis from an occult cancer, a gestational choriocarcinoma from previous pregnancy or have arisen from a concurrent hydatidiform mole genotyping of DNA from the tissue and normal surrounding placenta was performed [8]. Briefly, 5 ng of DNA from the normal placenta and tumour tissue were amplified with a panel of primers for 15 short tandem repeat (STR) loci on 13 autosomes and the amelogenin locus using an AmpFlSTR Identifiler Plus kit (Applied Biosystems, Warrington, UK). PCR products were resolved by capillary electrophoresis using an ABI 3100 Genetic Analyser and genotypes determined using GeneMapper version 5.0 software (Applied Biosystems).

### Whole genome sequencing

Genomic DNA libraries were prepared following Illumina’s (Illumina, San Diego, CA) suggested protocol and sequenced by Hiseq X Ten (Illumina) with 150PE. Somatic variants were identified using GATK Best Practices Pipeline. After Illumina sequencing, all produced FASTQ reads were quality-checked and trimmed with FastQC (version 0.11.2 http://www.bioinformatics.babraham.ac.uk/projects/fastqc/) and Trimmomatic (version 0.33). The average coverage of each base in the genome was 36.31 for the tumours and 23.11 for the placenta. The Q30 (%) was 91.20%. Sequencing reads were aligned to human genome hg19 with the BWA MEM software for both tumour and normal samples. PCR duplications of each BAM file were marked with Picard software (version 1.103 https://broadinstitute.github.io/picard/). The BAM files were locally realigned, and the base quality scores were recalibrated with GATK (version 3.1). Single nucleotide variants and Indels were detected using MuTect (version 1.1.6) and Strelka (version 1.0.14) respectively. All the somatic variants were validated with Integrative Genomics Viewer and annotated with Variant Effect Predictor (version 83).

### Methylation array hybridization

FFPE-derived DNA from both the tumour and placenta tissue were hybridized to the Illumina Infinium Methylation EPIC (EPIC) arrays. Bisulphite conversion was performed according to the manufacturer’s recommendations for the Illumina Infinium Assay (EZ DNA methylation kit, ZYMO, Orange, CA) and subjected to the Infinium FFPE QC and restoration kit prior to hybridisation.

### Data filtering and analysis of methylation signals

Before analysing the data, possible sources of technical biases that could influence results were excluded. We applied signal background subtraction using default control probes in BeadStudio (version 2011.1_Infinium HD). We discarded probes with a detection *P*-value > 0.01, containing single nucleotide polymorphisms (SNPs) within the interrogation or extension base as well as those with potential cross-reaction due to multiple sequence homologies. We also excluded probes that lacked signal values in one or more of the DNA samples analysed and that mapped to the X & Y chromosomes. In total 772,399 probes were investigated. For the analysis of known imprinted ubiquitous differentially methylated regions (DMRs), probes mapping to the intervals identified by Hernandez-Mora et al. were directly examined [9]. The names of each region are in accordance with the recently recommended nomenclature for clinical reporting of imprinted methylation profiles [10]. For placenta-specific imprinted DMRs, only probes mapping to regions with confirmed allelic methylation were interrogated [11]. The common regions of CpG island Methylator Phenotype (CIMP) were taken from Martin-Trujillo et al. [12]. In-house bioinformatics R scripts were utilized for statistics to identify loci with different methylation profiles between the two samples and to compare the FFPE-derived DNA methylation profiles with those obtained from high-molecular weight DNAs extracted directly from paired first trimester chorionic villous samples and term biopsies (GEO repository GSE121056) [13]. The data from the FFPE placenta and tumour are available in the GEO repository with the accession number GSE125386.

### Bisulphite methylation analyses

For confirmation PCR analyses, loci with the greatest significant difference identified using the EPIC arrays were targeted using standard bisulphite PCR. Furthermore, these and those analysed by pyrosequencing, had underlying converted sequences compatible with optimal primer design resulting in short products incorporating numerous CpG dinucleotides to ensure efficient amplification in FFPE-degraded input DNA.

Allelic PCR: Approximately 1 μg FFPE-derived DNA was converted using the EZ DNA Methylation-Gold kit (Zymo) following manufacturer’s instructions for short incubation times to avoid additional fragmentation of the DNA during the treatment. Approximately 5 μl of bisulphite converted DNA was used in each amplification reaction using Immolase Taq polymerase (Bioline, UK) for 45 cycles and the resulting PCR product sub-cloned into pGEM-T easy vector (Promega) for sequencing (for primer sequences see Additional file 1: Table S1).

Pyrosequencing: Standard bisulphite PCR was used to amplify 50 ng of bisulphite converted DNA with the exception that one primer was biotinylated (for primer sequence see Additional file 1: Table S1). The entire biotinylated PCR product (diluted to 40 μl) was mixed with 38 μl of Binding buffer and 2 μl (10 mg/ml) streptavidin-coated polystyrene beads. After incubation at 65 °C, DNA was denatured with 50 μl 0.5 M NaOH. The single-stranded DNA was hybridized to 40-pmol sequencing primers dissolved in 11 μl annealing buffer at 90 °C. For sequencing, a primer was designed to the opposite strand to the biotinylated primer used in the PCR reaction. The pyrosequencing reaction was carried out on a PyroMark Q96 instrument. The peak heights were determined using Pyro Q-CpG1.0.9 software (Biotage, Sweden).

## Results

### Fluorescent microsatellite genotyping analysis

The fluorescent microsatellite genotyping analysis (Additional file 2: Table S2) demonstrated the tumour to have the same genotype as the healthy placenta, confirming the identity of the tumour as a non-molar gestational choriocarcinoma arising in the current pregnancy. Furthermore, these results were endorsed by analysis of 65 highly informative SNPs present on the Illumina EPIC methylation array.

### Whole genome sequencing

Whole genome sequencing did not demonstrate any mutations of the common cancer associated oncogenes or any other driver mutations. The overall mutational load was extremely low, both in the number of detected mutations and also the proportion of the DNA prepared from the malignant cells that carry any of these apparent mutations (Table 1). Overall the results indicate that within this tumour the mutational load was extremely low and unlikely to be of biological significance.

**Table 1:** Results of WGS of the choriocarcinoma and placenta

Gene_Name	Start Position	End Position	Variant Classification	Ref	Alt	cDNA Change	Protein Change	Tumour mutant frequency	Normal mutant frequency
GIGYF2	233712210	233712230	Inframe deletion	CAGCAGCAGCAGCTGCCACAG	-	c.3689_3709 delTGCCACAGCA GCAGCAGCAGC	p.Leu1230_ Gln1236del	0.111	0
LOC101929543	170064859	170064859	missense_variant	G	T	c.460G>T	p.Gly154Cys	0.108	0
LOC101928951	143582475	143582475	missense_variant	C	T	c.698C>T	p.Ala233Val	0.138	0
ANKRD20A5P	14188003	14188003	splice_acceptor_variant	G	A	n.791-1G>A	.	0.16	0
NBPF9	144823176	144823176	missense_variant	C	A	c.1077C>A	p.Pro360Thr	0.184	0.021
C6	41199959	41199959	missense_variant	G	T	c.356C>A	p.Ala119Glu	0.167	0
IQSEC3	176139	176139	missense_variant	A	T	c.91A>T	p.Thr31Ser	0.098	0
IGFBP3	45960645	45960645	missense_variant	G	C	c.95C>G	p.Ala32Gly	0.109	0.021

The results of the WGS analysis from the tumour and placenta are shown. The results indicate a very low level of mutation within the tumour. There were no mutations in any oncogenes. The mutations noted are unlikely to be of biological importance

### Methylation studies

**Fig. 2:**
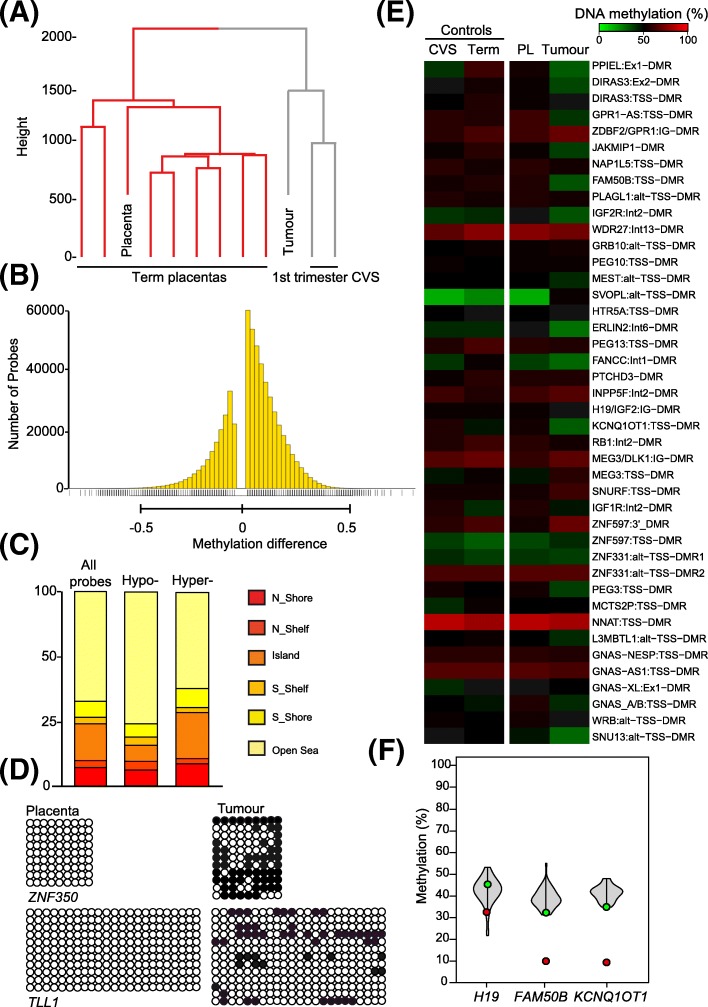
Characterization of DNA methylation in the placenta and tumour using the Illumina EPIC methylation array. **a** Hierarchical clustering of global methylation for placenta-tumour paired samples with 8 term placenta and 2 first trimester CVS samples. **b** Bar graph of the distribution of the hypo- (left side) and hypermethylated (right side) probes with a difference greater that −/+ 2.5% (0.025ß) when comparing the tumour and paired placenta. **c** Classification of probes with differential methylation according to genomic location. The bar chart illustrates probe enrichment classified by Illumina Infinium annotation. **d** Bisulphite confirmation of methylation difference between tumour and paired placenta samples. Each circle represents a single CpG dinucleotide on a DNA strand. (•) Methylated cytosine, (o) unmethylated cytosine. Each row corresponds to an individual cloned sequence. **e** Heatmap of the ubiquitous imprinted DMR probes in control first trimester CVS and term placenta biopsies as well as the tumour and paired placenta samples. The values represent the average of all probes mapping to each region. **f** Methylation levels of ubiquitous imprinted DMRs in the placenta (green dot), tumour (red dot) and controls (violin plots *n* = 16) quantified by pyrosequencing

In contrast the epigenetic studies demonstrated that the pattern of methylation is markedly different between the tumour, the surrounding normal placenta or reference unrelated term placental samples. To determine whether there was any resemblance of the methylation profile in the tumour with early placental samples we obtained DNA from 12-week chorionic villus biopsies. Interestingly, using both partitioning and hierarchical clustering, we observed that the tumour has a genome-wide methylation profile resembling the first trimester chorionic villous samples, whilst the placenta is located in a separate branch with normal term controls (Fig. 2a). To identify specific loci that are differentially methylated between the tumour and placenta we performed an unsupervised search between the two samples. This revealed 177816 hypermethylated positions and 405383 hypomethylated by −/+ 2.5% (0.025ß), the accepted detection resolution of the Infinium assays [14]. Of these 336 differed by more than 50% (0.5ß) (Fig. 2b). Subsequently we performed bumphunter analysis to identify multiple probe clusters of which 2645 were hypermethylated and 3365 hypomethylated (Additional file 3: Table S3). In general, hypermethylated intervals were larger (top 200 candidates; mean length 877 bp SD 594 bp, containing on average 10.4 probes) than hypomethylated regions (top 200 candidates; mean length 257 bp SD 325 bp containing on average 3.5 probes), however these differences may reflect a bias in assay design since probes in intergenic regions are underrepresented. When the genomic location of the differentially methylated probes is taken into consideration, “CpG island” probes are clearly less prone to be hypomethylated in the tumour with respect to the placenta, while “CpG islands, shelf and shores” are hypermethylated (Fig. 2c). To validate the methylation profiles obtained from the EPIC array comparisons we performed bisulphite PCR and sub-cloning of two regions. In each case we confirm the profile observed, with *TLL1* and *ZNF350* being more methylated in the tumour than placenta (Fig. 2d).

Next, we analysed the methylation profiles at imprinted differentially methylated regions (DMRs) since these are closely linked to both placental development and tumourigenesis [15]. Of the 40 ubiquitous imprinted DMRs present on the EPIC platform loss-of-methylation (LOM) was far more frequent than gains-of-methylation (GOM) with 18 regions comparable between the two samples, 16 modestly hypomethylated and 6 hypermethylated in the tumour (Fig. 2e, Additional file 4: Table S4). One region of GOM, at the paternally-methylated *ZDBF2* DMR is as a direct consequence of hypomethylation of the adjacent maternally-methylated *GPR1-AS1* DMR which is responsible for appropriate allelic methylation at this domain in a hierarchical manner [16]. To ensure the EPIC array data truly reflects the methylation profile at imprinted DMRs, we performed pyrosequencing for the *H19*, *KCNQ1OT1,* and *FAM50B* regions which confirmed that the tumour sample is 22–24% less methylated than the corresponding placenta (Fig. 2f). We subsequently extended this analysis to placenta-specific imprinted DMRs. Of the samples with appropriate methylation in the placenta samples (these are polymorphic epialleles so we only analysed those with a pattern consistent with allelic methylation in the normal placenta sample), 97 regions were comparable between the two samples and 69 modestly hypomethylated (Additional file 5: Table S5), with the levels at the *CMTM3* and *GLIS3* DMRs confirmed by pyrosequencing (Additional file 7: Figure S1A and B).

Finally, potential changes in methylation associated with the tumourigenic process have previously been reported in samples with global hypomethylation. Widespread CpG island promoter hypermethylation, also referred to as CpG island methylator phenotype (CIMP), has been reported for many tumour types [17]. With the exception of *CRABP1*, the analysis of 23 CIMP regions revealed very little tumour-associated hypermethylation (Additional file 7: Figure S1C, Additional file 6: Table S6) suggesting that CIMP may not be a universal phenomenon in gestational choriocarcinomas.

## Discussion

To date there has been relatively little work examining the potential role of genetic changes in the pathogenesis of gestational tumours. However, recent data from targeted sequencing of 6 cases of gestational choriocarcinoma, examining 637 cancer related genes, has indicated an extremely low level of somatic mutation [18]. Similarly targeted sequencing of cases of the PSTT and ETT, gestational malignancies that arise slightly later in placental development, have also demonstrated very low levels of mutation and a lack of any repetitive or driver mutations [19].

Intra-placental gestational choriocarcinomas, in which the tumour is identified in the placenta during pregnancy or at delivery are extremely rare but represent a very early form of gestational malignancy [7]. As a result, any genetic changes identified in an intra-placental gestational choriocarcinoma are likely to represent the earliest changes associated with tumour development.

In this report, we have aimed to more fully characterise the genetic and epigenetic changes in a case of intra-placental gestational choriocarcinoma. We first confirmed the gestational origin of the malignancy found within the placenta by microsatellite genotyping analysis, the results shown in Additional file 2: Table S2 demonstrate identical results at all 16 loci. This result excludes the possibility that the tumour in the placenta could either have been a metastasis from an occult primary cancer or have arisen from a prior conception or concurrent molar pregnancy.

Further analysis by whole genome sequencing of the tumour with comparison to the neighbouring healthy placenta failed to demonstrate any mutations in any established oncogenes or any appreciable mutations elsewhere in the genome as shown in Table 1. This finding, a lack of any significant mutation in this case, supports the earlier genetically bland findings obtained from sequencing of a limited number of genes in a panel of trophoblast tumours [18, 19]. A number of paediatric malignancies, which each share a relatively short oncogenesis timeframe, similarly have very low overall levels of mutations. In these other rare diagnoses, the cells gain their malignant phenotype from single driver mutations in the case of infantile acute lymphoblastic leukaemia and paediatric rhabdoid tumours or epigenetic changes alone in CIMP-positive ependymoma [20–22].

It is apparent that the time frame for the development of malignancy in gestational choriocarcinoma is even shorter than for these paediatric malignancies. We have previously questioned whether accumulation of mutations, as in the common epithelial malignancies, could be the route to oncogenesis of this rare malignancy [6]. The demonstration of a lack of significant mutation in this case analysed by WGS and in other cases of trophoblast tumours previously analysed by limited genomic profiling would support the argument that gestational choriocarcinoma does not appear to be a mutation driven malignancy [18, 19].

Normal early trophoblast cells appear to share many characteristics of malignant choriocarcinoma cells including rapid proliferation, hCG production, an ability to invade into other tissues, stimulation of angiogenesis and also the extreme sensitivity to DNA damaging chemotherapy [23, 24]. As a result, it has been hypothesised that gestational choriocarcinoma may occur not as a result of genetic change but via the inappropriate persistence of normally transient primitive trophoblast cells, that are unable to either mature or undergo apoptosis. As a result, these cells could be locked in a frozen developmental state of an early trophoblast cell’s and retain much of that cells normal phenotypically malignant phenotype [6].

In the absence of a mutational cause for oncogenesis we examined the methylation status of the tumour DNA. Normal placental development is associated with wide spread epigenetic changes, the transition from first to third trimester being associated with increasing hypermethylation [25–27]. Examination of the methylation profile using high-throughput arrays of the tumour and normal placenta in the current case showed the methylation patterns of the tumour and the mature placenta differ significantly (Fig. 2). The tumour sample was hypomethylated compared to the placenta at most ubiquitous and placenta imprinted DMRs. Despite the differences detected using EPIC methylation arrays being modest, validation using quantitative pyrosequencing revealed a similar degree of hypomethylation at three ubiquitous and two placenta specific imprinted DMRs. Hierarchical clustering demonstrated the tumour to cluster with first trimester chorionic villous samples while the placenta clustered with the term controls. This demonstration that the methylation profile of the tumour is close to that of a first trimester placenta rather than a mature placenta supports the hypothesis that gestational choriocarcinoma arises from an early trophoblast cell [23].

The biological processes involved in early pregnancy share several phenotypic hallmarks of cancer. After undergoing epithelial-to-mesenchymal transition, placental cells invade and migrate within the endometrium whilst evading maternal immune system. The similarities between placenta development and cancer also extends to their unusual epigenomes. Recently, Nordor and colleagues reported that loci undergoing widespread hypomethylation in placenta were of similar size and location as those distinguishing tumours from matched normal tissues [28].

In addition to the widespread intergenic hypomethylation evident in this case of intra-placental gestational choriocarcinoma, many promoter CpG islands underwent hypermethylation. Promoter hypermethylation of tumour-suppressor genes is a frequent and key event in tumorigenesis. Of the promoters gaining methylation in our sample, many have already been shown to have tumour suppressor activity, including RORa, FAN1, PRDM1, CYGB, L3MBTL4, EPB41L3, with ZNF471 and ZNF671 being specifically silenced by methylation [29, 30]. Strikingly, like ZNF471 and ZNF671, 27 of the top 200 hypermethylated genes identified in this study map to zinc-finger genes, with 75% mapping to the Chr19q41–43 cluster, suggesting that altered expression of these DNA-binding proteins maybe involved in the development of gestational choriocarcinoma.

The concept that the onset of malignancy can cause a halt in the normal developmental pathways and prevent onset of the normal processes of apoptosis is well established in the lymphoid malignancies [31]. The impact of stopping the normal development of the trophoblast cell at the time of transformation to the choriocarcinoma cell, may explain the extreme sensitivity of these malignant cells to DNA damaging chemotherapy drugs. This characteristic of normal early trophoblast cells is exploited in the medical management of ectopic pregnancy which is effectively treated with low doses of the chemotherapy agent methotrexate [32].

The ability of methylation changes alone to result in oncogenesis has already been demonstrated in CIMP-positive ependymoma, a rare malignancy occurring in children. Studies looking at a panel of these tumours have demonstrated that there are only overall very low levels of mutations with no discernable patterns or driver mutations [22]. In this form of ependymoma it appears that the production of the malignant phenotype occurs via epigenetic changes including transcriptional silencing of key developmental genes.

It is difficult to draw firm conclusions based on the genome analysis of FFPE material from a single case of intraplacental gestational choriocarcinoma. However, our data suggest that the malignant phenotype in this diagnosis, as in ependymomas, may be based on epigenetic rather than genetic changes. We predict that the changes at imprinted loci lead to a block in the development of early trophoblast cells at a stage where their phenotype is naturally close to that of a malignant cell. The importance of the differing basic physiology of the cell origin in determining the sensitivity to cytotoxic chemotherapy is clearly demonstrated in these two malignancies that occur without DNA mutations. Whilst gestational choriocarcinoma is extremely sensitive to chemotherapy and highly curable, CIMP ependymoma which is also methylation driven and has no mutations is extremely resistant to chemotherapy and carries a very poor prognosis [33].

## Conclusions

Based on the initial data available from this case it may be possible to suggest an important difference in the route to oncogenesis between gestational choriocarcinoma and other malignancies. Characteristically malignancies gain their malignant phenotype as a result of an aberrant genetic event. In contrast it appears that in gestational choriocarcinoma the cells do not gain the malignant phenotype, but they fail to lose the essentially malignant phenotype of a primitive trophoblast cell as a result of an aberrant event in the normal progression of methylation changes.

The natural history of gestational choriocarcinoma is extremely varied. The interval from pregnancy to presentation can be very short or can extend to greater than 20 years. Similarly, the clinical course can vary from a widely metastatic fast growing and life-threatening cancer to one that has more indolent behaviour presenting with repeated false positive pregnancy tests, but without clinical symptoms. It is likely that this width of clinical behaviour may be matched by differing patterns of methylation and gene expression. Further studies examining the role of methylation changes in other cases of trophoblastic tumours may provide confirmation of these observations and also a more detailed understanding of this route to oncogenesis.

## Additional files

Additional file 1: **Table S1** PCR primers. (XLSX 9 kb)

Additional file 2: **Table S2** Genotyping of DNA from placental villi and tumour tissue showing alleles identified at sixteen informative loci. (DOCX 14 kb)

Additional file 3: **Table S3** List of loci containing multiple Illumina EPIC probes identified by bumphunter analysis. (XLSX 721 kb)

Additional file 4: **Table S4** Methylation profiles of individual probes mapping within the ubiquitous, imprinted DMRs. (XLSX 62 kb)

Additional file 5: **Table S5** Methylation profiles of individual probes mapping within placenta specific imprinted DMRs. (XLSX 60 kb)

Additional file 6: **Table S6** Methylation profiles of individual probes mapping within CIMP domains. (XLSX 39 kb)

Additional file 7: **Figure S1** Methylation profiling specific loci using the Illumina EPIC methylation array. (A) Heatmap of the placenta-specific imprinted DMR probes in the tumour and paired placenta samples. (B) Methylation levels of *GLIS3* and *CMTM3*, two placenta-specific imprinted DMRs, in the placenta (green dot), tumour (red dot) and controls (violin plots *n* = 16) quantified by pyrosequencing. (C) Heatmap of the CIMP regions in the tumour and paired placenta samples. Notes, for (A) and (C) the array values represent the average of all probes mapping to each region. (PDF 434 kb)

## Data Availability

The DNA sequencing data is available on request. The methylation data has been uploaded to the GEO repository as detailed in the text.

## Abbreviations

CIMP: CpG island Methylator Phenotype
DMRs: Differentially methylated regions
ETT: Epithelioid trophoblastic tumour
FFPE: Formalin-fixed paraffin-embedded
GOM: Gains-of-methylation
hCG: Human chorionic gonadotrophin
LOM: Loss-of-methylation
PSTT: Placental site trophoblastic tumour
STR: Short tandem repeat
WGS: Whole genome sequencing
