# Supplementary material for: A case of intraplacental gestational choriocarcinoma; characterised by the methylation pattern of the early placenta and an absence of driver mutations
Source: BMC Cancer. 2019 Jul 29;19:744. doi: 10.1186/s12885-019-5906-8 (PMC6664587; doi:10.1186/s12885-019-5906-8)
Supplement: Supplementary file 2 — Table S2 Genotyping of DNA from placental villi and tumour tissue showing alleles identified at sixteen informative loci. (DOCX 14 kb) [file 12885_2019_5906_MOESM2_ESM.docx]

| **Locus** | **Alleles** | |
| --- | --- | --- |
|  | **Placental Villi** | **Tumour Tissue** |
| **D8S1179** | 14 - 16 | 14 - 16 |
| **D21S11** | 28 - 31.2 | 28 - 31.2 |
| **D7S820** | 10 - 11 | 10 - 11 |
| **CSF1PO** | 10 - 11 | 10 - 11 |
| **D3S1358** | 14 - 15 | 14 - 15 |
| **THO1** | 6 - 10 | 6 - 10 |
| **D13S317** | 9 - 12 | 9 - 12 |
| **D16S539** | 11 - 12 | 11 - 12 |
| **D2S1338** | 17 - 22 | 17 - 22 |
| **D19S433** | 15 - 15.2 | 15 - 15.2 |
| **vWA** | 17 - 18 | 17 - 18 |
| **TPOX** | 8 - 9 | 8 - 9 |
| **D18S51** | 16 - 19 | 16 - 19 |
| **AMEL** | X | X |
| **D5S818** | 11 - 12 | 11 - 12 |
| **FGA** | 20 - 26 | 20 - 26 |

**Table S2: Genotyping of placental**

**villi and tumour tissue.**
