# Supplementary figures and images for: A case of intraplacental gestational choriocarcinoma; characterised by the methylation pattern of the early placenta and an absence of driver mutations
Source: BMC Cancer. 2019 Jul 29;19:744. doi: 10.1186/s12885-019-5906-8 (PMC6664587; doi:10.1186/s12885-019-5906-8)

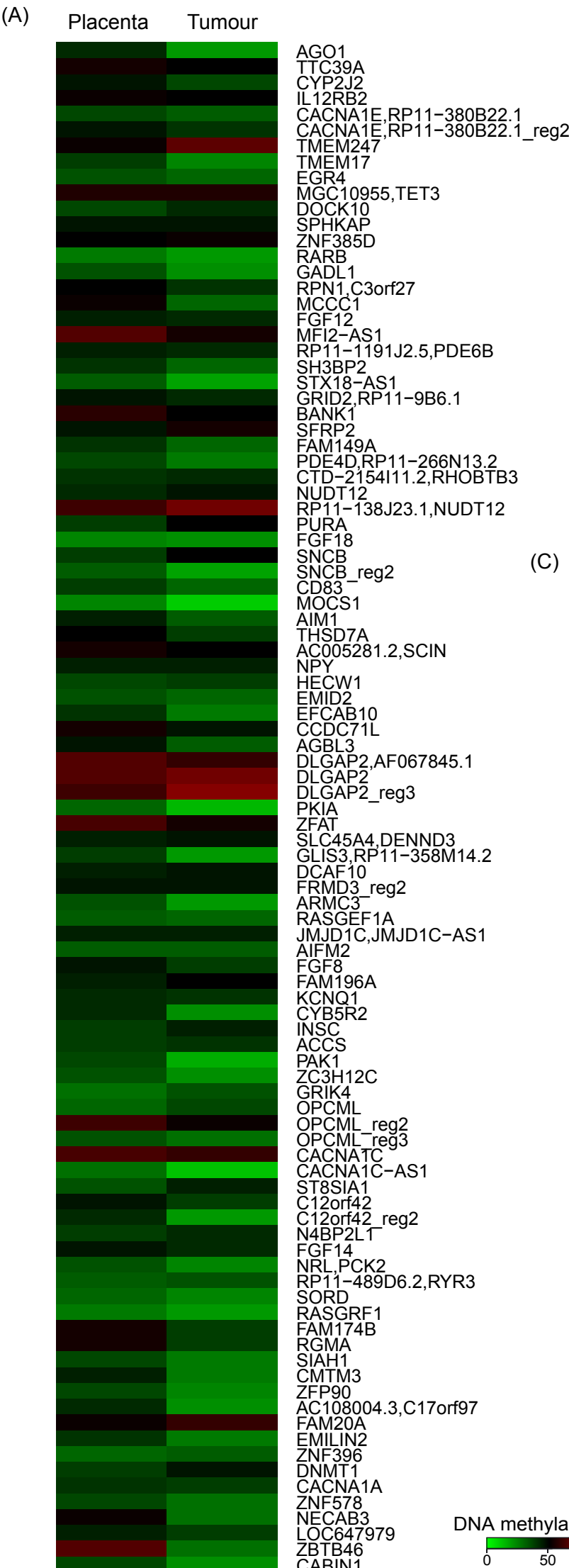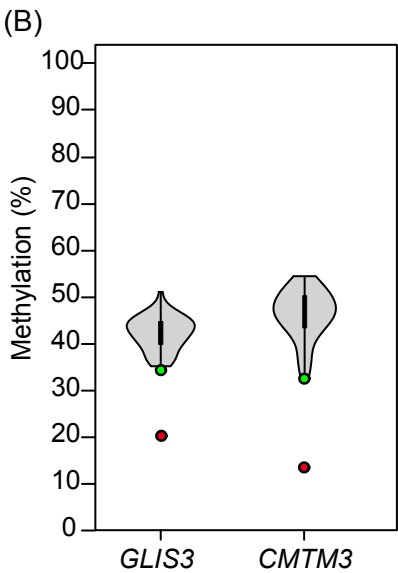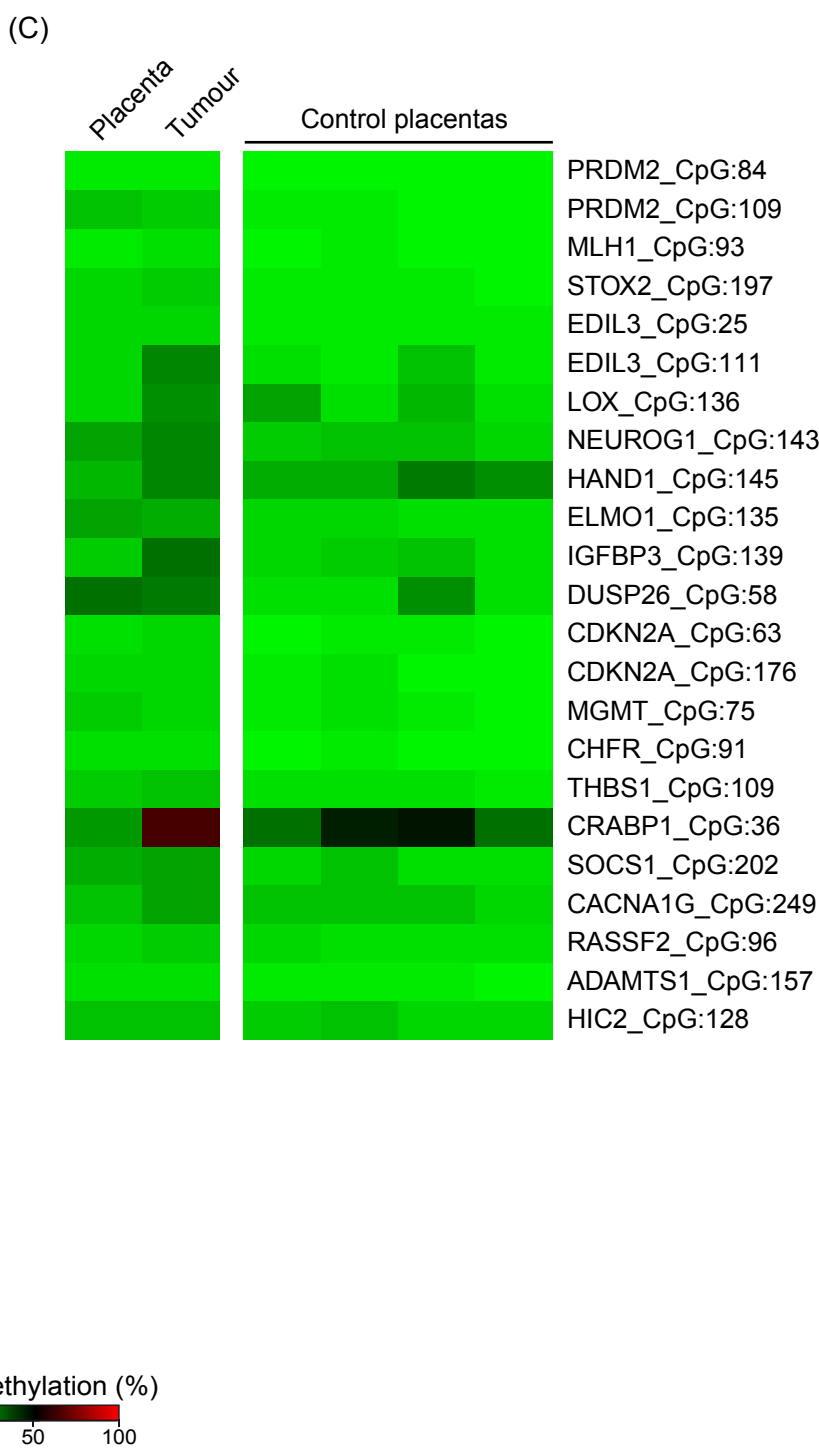

Supplement: Supplementary file 7 — Figure S1 Methylation profiling specific loci using the Illumina EPIC methylation array. (A) Heatmap of the placenta-specific imprinted DMR probes in the tumour and paired placenta samples. (B) Methylation levels of GLIS3 and CMTM3, two placenta-specific imprinted DMRs, in the placenta (green dot), tumour (red dot) and controls (violin plots n = 16) quantified by pyrosequencing. (C) Heatmap of the CIMP regions in the tumour and paired placenta samples. Notes, for (A) and (C) the array values represent the average of all probes mapping to each region. (PDF 434 kb) [file 12885_2019_5906_MOESM7_ESM.pdf]
